# ‘We identify, discuss, act and promise to prevent similar deaths’: a qualitative study of Ethiopia's Maternal Death Surveillance and Response system

**DOI:** 10.1136/bmjgh-2016-000199

**Published:** 2017-03-14

**Authors:** Berhanu Abebe, Joanna Busza, Azmach Hadush, Abdurehman Usmael, Amsalu Belew Zeleke, Sahle Sita, Solomon Hailu, Wendy J Graham

**Affiliations:** 1 MCH Department, WHO Ethiopia Country Office, Addis Ababa, Ethiopia; 2 Department of Population Health, London School of Hygiene and Tropical Medicine, London, UK; 3 IMMPACT, University of Aberdeen, Aberdeen, UK

## Abstract

**Introduction:**

Ethiopia introduced national Maternal Death Surveillance and Response (MDSR) in 2013 and is among the first sub-Saharan African countries to capture data on facility-based and community-based maternal deaths. We interviewed frontline MDSR implementers about their experiences of the first 2 years of MDSR, including perceptions of its introduction and outcomes for health services.

**Methods:**

We conducted a qualitative case study in 4 zones in the largest regions, interviewing 69 key informants from regional, zonal, district and facility levels.

**Results:**

A defining feature of Ethiopia's MDSR system is its integration within existing disease surveillance, with both benefits and challenges. Facilitators of the system's introduction were strong political support, alignment with broader health strategies and strong links across health system departments. Barriers included confusion around new responsibilities, high staff turnover and fear of legal repercussions. Stakeholders believed MDSR increased confidence in using local data to improve maternal health services and enhanced communication across the health system.

**Conclusions:**

MDSR systems take time to establish, encountering challenges in early implementation. Ensuring MDSR has a clear purpose, explicitly defined roles and responsibilities, and adequate supervisory support from the start will ensure it becomes embedded within the health system as routine practice rather than perceived as a stand-alone system. Countries planning to adopt or extend MDSR can learn from Ethiopia's experience, particularly the decision to make maternal mortality a weekly reportable condition within Public Health Emergency Management.

**Key questions**

**What is already known about this topic?**

- Maternal Death Surveillance and Response (MDSR) systems are recommended to improve empirical data on maternal mortality and identify effective interventions.
- An increasing number of developing countries are introducing MDSR systems as part of their strategies to reduce maternal mortality.
- Ethiopia is one of the first sub-Saharan African countries implementing MDSR in facilities and communities, providing useful lessons for other settings.

**What are the new findings?**

- Integrating maternal mortality into existing national disease surveillance is a unique approach to MDSR that brings both benefits and challenges.
- MDSR functionality depends on political commitment, clearly defined roles and responsibilities, and alignment with broader health policies.
- Improved confidence in data reliability, stronger motivation to target determinants of maternal mortality and better communication across the health system were seen to be early successes.
- High staff turnover, delayed distribution of data collection tools and fear of repercussions for reporting maternal deaths threaten MDSR sustainability.

**Recommendations for policy**

- Learning lessons from Ethiopia's early implementation of MDSR can help inform its scale-up as well as its introduction in other countries.
- Understanding MDSR strengths and weaknesses during its start-up phase can help identify measures to maximise early gains, address risks to the system and improve its sustainability.

## Introduction

The inclusion of Maternal Death Surveillance and Response (MDSR) in a country's health strategy is considered a key component of efforts to reduce maternal mortality.^1^ The WHO has developed guidance for local adaptation and tracks implementation progress across countries,^2^ and initiatives such as the Global Strategy for Women's and Children's Health have endorsed the role of MDSR systems in informing maternal health policy.^3^

A well-functioning MDSR system supports a continuous process of identifying maternal deaths, collecting detailed information on their causes and determinants, and analysing these data to select responses that will prevent similar events in future.^4^ At facility level, actions address provider skills, medical supplies, referral mechanisms and community relations,^5^ ^6^ while at higher levels the focus is on interpreting patterns and trends in maternal deaths and targeting resources to reduce inequities, improve public awareness and health-seeking, and strengthen the health architecture.^7^ ^8^

Fully functioning MDSR systems are primarily found in developed countries such as the UK, where the maternal mortality ratio (MMR) is already low and every maternal death can be individually scrutinised through a confidential inquiry.^9^ MDSR systems in resource-poor settings are increasing and most focus on facility-based deaths, for example, South Africa and Malaysia.^8^ ^10^ ^11^ Facility-based systems are most appropriate where high proportions of deliveries occur in facilities but they are less able to capture ‘upstream’ determinants of maternal death related to nutrition, underlying health and broader socioeconomic conditions.^12^ The challenge, therefore, is to create an MDSR system in which both facility-based and community-based deaths are included, and sufficient data captured for each case to allow for interpretation across all deaths.^13^ This is particularly important where a significant proportion of women deliver at home.^14^

Ethiopia is among the first countries in sub-Saharan Africa to introduce an MDSR system designed to be comprehensive. With a population of over 90 million,^15^ over 70% of deliveries occurring without skilled birth attendance^14^ and an estimated MMR of 353,^16^ this has proved an ambitious endeavour but one with high levels of political commitment. The national MDSR system was launched in May 2013 by the Minister of Health, following over a year of preparatory work including formation of a task force, development of national guidance and training curriculum. In 2014, MDSR was formally included in Ethiopia's integrated disease surveillance structure, known as Public Health Emergency Management (PHEM). Maternal death became the 21st weekly reportable condition.^17^

A phased approach was taken to implementation, beginning with the four largest regions (Amhara, Oromia, Southern Nations, Nationalities and People's Region (SNNPR) and Tigray) and three city administrations (Addis Ababa, Dire Dawa and Harari) that together represent 90% of the population.^14^ In late 2015, Ethiopia began rolling out MDSR to the remaining four pastoralist regions. It is thus in the process of transitioning from a setting-up phase to routine implementation.

**Figure 1:**
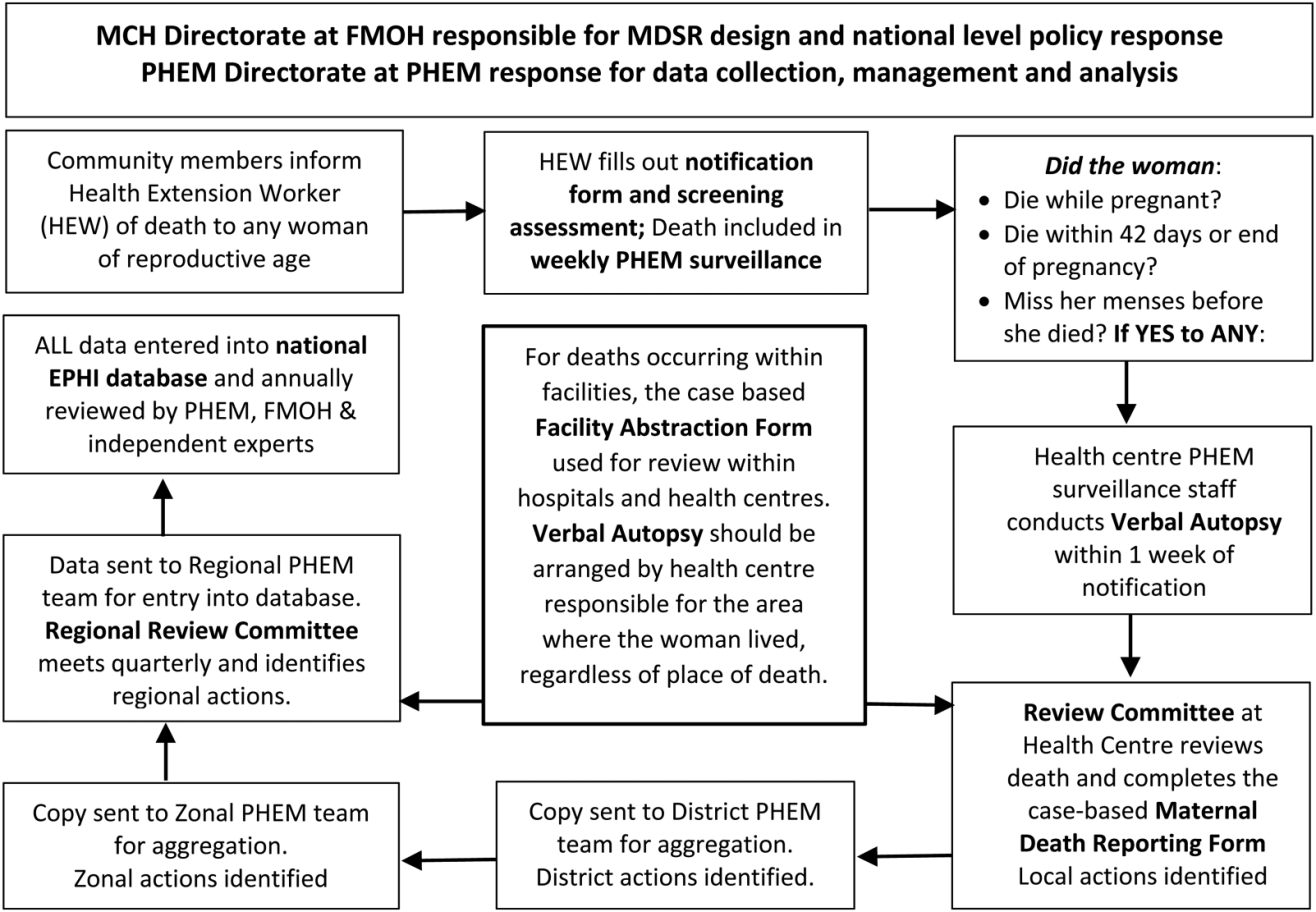
Ethiopia's MDSR system structure and data flow. EPHI, Ethiopian Public Health Institute; FMOH, Federal Ministry of Health; MCH, Maternal and Child Health; MDSR, Maternal Death Surveillance and Response; PHEM, Public Health Emergency Management.

Figure 1 provides a brief overview of Ethiopia's MDSR system's structure, including expected data flow and key actors involved in reporting, analysing and interpreting maternal mortality data at the various levels of the national health system.

This paper presents the results of a qualitative policy assessment of stakeholders' perceptions of the process and outcomes of Ethiopia's MDSR system following the first 2 years of implementation. Findings draw on data collected by the Evidence for Action programme (E4A), a maternal and newborn health initiative delivered through the WHO country office and supported by the University of Aberdeen from 2012 to 2016. E4A contributed to the design of national MDSR reporting instruments, training programme, national and regional databases, and provided seven technical assistants (TAs) to support facilities, district, zonal and regional health offices as well as central data management.

## Methods

### Study design

Two years after MDSR introduction, E4A collected data in the first four zones (one per region) where it provided technical support. We used a qualitative case study approach, interviewing health managers from facilities (health centres, district and referral hospitals), and public health professionals at district, zonal and regional levels. We focused on staff at the frontline who were responsible for delivering MDSR rather than national-level staff who originally designed it. The study was conducted as a rapid assessment, over a period of 8 weeks in July to September 2015.

The aim of the study was to document and assess the introduction of MDSR and its first 2 years of implementation in Ethiopia in order to identify practical lessons that might inform MDSR in other settings as well as its scale-up across Ethiopia. Research questions addressed both process and early outcomes of MDSR, as follows: (1) What were the facilitators and barriers experienced by frontline health systems staff when tasked with introducing a standardised national MDSR system? (2) How do different stakeholders perceive the current status of the MDSR system including whether and how it contributes to improving maternal health?

Study sites were West Gojjam Zone in Amhara Region (including the city of Bahir Dar, which is surrounded by West Gojjam), West Arsi Zone in Oromia, Guraghe Zone in SNNPR and Southern Zone in Tigray. Table 1 summarises some key features of each zone.

**Table 1:** Study zones

Region	Study zone	Population	Number of districts	Number of hospitals	Number of health centres
Amhara	West Gojjam	2 560 131	16	5	102
Oromia	West Arsi	2 335 512	14	4	79
SNNPR	Guraghe	1 631 499	15	5	71
Tigray	South Tigray	713 275	8	3	32

Sources of information: Regional Health Bureau.

SNNPR, Southern Nations, Nationalities and People's Region.

### Sampling and recruitment

We interviewed all four regional MDSR focal persons (housed in the Maternal and Child Health (MCH) directorate). In each zone, one district was selected based on having both rural and urban catchment areas and all zonal-level public health staff engaged in implementing MDSR were interviewed, including representatives in the MCH and PHEM departments. We further interviewed one referral or district hospital director per zone, as well as on-duty managers from at least one remote and one easily accessible health centre.

Table 2 shows distribution across zones of the 69 respondents (32 individual and 14 group interviews). Group interviews included 2–4 participants, and were held when respondents requested that they participate together to save time. Where PHEM and MCH focal persons shared an office or were available on the same site, they preferred to be interviewed as a group.

**Table 2:** Sampling

	Amhara	Oromia	SNNPR	Tigray	Total respondents
Individual interview	7	6	12	6	31
Group interview	8	9	10	11	38
Total respondents	15	15	22	17	69

SNNPR, Southern Nations, Nationalities and People's Region.

The TA employed by E4A in each region was responsible for logistical arrangements. The first author (BA) conducted data collection. Usually based in Addis Ababa, he had no prior contact with respondents or involvement in supporting MDSR in any of the study sites. Regional TAs made initial contact by phone or in person in order to inform respondents of the purpose of the study and invite them to participate. An appointment time and location were arranged, and the local TA accompanied BA to the interview but left prior to data collection.

### Data collection and management

Interviews took place in a private location, usually a meeting room or spare office at the respondents' workplace. A semistructured topic guide was used (available as an online supplementary file), slightly adapted for each type of respondent. The interviews asked about MDSR introduction, initial facilitators and barriers, and current challenges and/or benefits. Respondents were also asked to comment on their concerns regarding the programme, any recommendations for its future improvement and specifically whether MDSR had affected key areas of quality of care, for example, transportation, provider skill and continuity of medical supply. All interviews were conducted in Amharic (the national language of Ethiopia), audio-recorded and transcribed into English by four research assistants contracted specifically for this study. Interviews took between 30 min and 2 hours, and respondents were not compensated.

10.1136/bmjgh-2016-000199.supp1 supplementary file

### Ethical approval

Ethical approval for this study was obtained from the London School of Hygiene and Tropical Medicine, the University of Aberdeen, and Regional Health Bureau Ethical Review Boards in Amhara, Oromia, SNNPR and Tigray. Written informed consent was obtained from all respondents, including for audio-recording and using excerpts in publications and reports. Respondents received an information sheet with contact details for the study team, and their confidentiality was assured. Identifying names of individuals and locations (such as specific health centres or hospitals) were removed during transcription.

### Analysis

Thematic content analysis was conducted following data familiarisation (reading and re-reading of the transcripts). Analysis was conducted on translated English transcripts, with reference to the Amharic digital recordings for clarification. The coding framework followed the topic guide and text was coded into broad themes defined a priori: facilitators, barriers, challenges and effects of the first 2 years of MDSR in Ethiopia according to public health sector staff and medical providers. The second author (JB) conducted initial analysis, refined through discussion with other team members. No software was used.

Additional subthemes were identified as they emerged from the data and added to the coding frame, such as concerns about staff turnover and the persistence of fears of repercussions for admitting to maternal deaths. Analysis specifically examined differences between cadres of the health system (regional or zonal health bureau policymakers vs providers in hospitals and health centres and Maternal, Newborn and Child Health (MNCH) experts vs PHEM staff). Differences by region were not investigated as the study aimed to capture a national ‘snapshot’ of Ethiopia's MDSR experience and to avoid raising political sensitivities around competition between regions.

## Results

This paper presents common experiences of early MDSR implementation in all four study zones. Similarities in facilitators, barriers, challenges and effects are illustrated by direct excerpts from the interviews, which are identified by location and respondent's role if there were at least four or more individuals in that category. For respondents who might inadvertently be identified by their role (ie, Regional Health Bureau Deputy Head) only level of the health system is provided without geographical distinction.

Findings are provided in two sections centred on (1) the process of MDSR introduction and (2) perceptions of MDSR outcomes after 2 years. Within these topic areas, data are presented on respondents' understandings of facilitators and barriers to initiating and sustaining MDSR, views on its strengths and weaknesses, observations of its likely effects, and suggestions for improvement in future.

### Process: How was MDSR's introduction experienced on the ground?

#### MDSR received political commitment and aligned with existing national health goals

*All* respondents mentioned Ethiopia's high-level political commitment to reducing maternal mortality as a driving force behind MDSR. They referred to the Federal Ministry of Health's (FMOH) emphasis on meeting Millennium Development Goals (MDG) 4 and 5, national pride in and media attention to achievement of MDG 4 ahead of schedule, and prioritisation of maternal heath following insufficient progress on MDG 5a.

Clear messages from the Ministry imbued MDSR with a sense of prioritisation and urgency, and respondents thus felt obliged to deliver MDSR as part of national plans to accelerate reductions in maternal mortality.

Maternal death was a political issue in government, so the district gave high attention to maternal death and everyone was politically aware [of efforts] to achieve our goal. (Health Centre Director, Tigray)

The leadership played a great role. [There has been] linkage starting from the Federal Ministry of Health. (Regional Health Bureau MCH Team Leader)

My feeling was that the government gave due attention to maternal and child health services through creating MDSR program. (Health Centre Director, Oromia)

Leadership at lower levels also proved important, increasing pressure for results. Ethiopia's health system follows a clear hierarchical structure with standardised management procedures, including regularly scheduled ‘supportive supervision’ visits that are cascaded down the system and reported. In districts and zones, therefore, if MDSR was included as a topic for discussion during supervision, frontline staff understood the importance of the new programme and could not neglect its functions.

Professional commitment is one kind of support and the other is…strong supportive supervision from the district. (Zonal Health Bureau Manager, Amhara)

Similarly, clinical managers also needed to pay attention to MDSR for it to result in introduction of regular maternal death reviews and subsequent preventive action. Staff needed to be oriented to their responsibilities in the new system and to see how it fit with other work activities.

So what I did was share the reading materials about MDSR…[then I] established an MDSR committee and started our activity. We assigned one physician at the ward level to monitor MDSR. Then we created awareness for our midwives. Even we strengthened our [MDSR] awareness activities during training on TB or other related trainings. (Referral Hospital Director, Oromia)

Conversely, respondents described how weak supervision side-lined MDSR. Lack of guidance from above led to staff confusion on how to implement MDSR.

When something gets started there is confusion. What are we [supposed to] do exactly in MDSR and in the committee? We worried about that but through time…we understand it is very simple and it needs only supervision and attention. (Hospital health promotion coordinator, SNNPR)

A key reason why MDSR received high-level political support from the beginning was its close alignment with other MCH initiatives. Indeed, respondents highlighted that MDSR was explicitly mentioned in the country's main strategic policy guidance, the Health Sector Transformation Plans for 2010–2015 and 2016–2020.

This meant that when MDSR review committees identified determinants of local deaths, they could put into place responses that fit into existing and budgeted MCH annual plans, for example, improving transportation and referral systems, increasing community awareness, promoting institutional delivery, and strengthening service quality. MDSR was thus perceived as synergistic with ongoing activities, facilitating prioritisation rather than introducing new demands.

All activities are related to each other, mobilization for [facility] delivery brought changes through creating awareness. Even if the results are not directly from MDSR, it has effects on the skill of our staff. (District PHEM focal person, Oromia)

Before there was no ambulance at our hospital for referrals, but now we have an ambulance. MDSR is one part of our hospital activities. It cannot directly order an ambulance, but pushes the management to solve the problem. (Hospital provider, Oromia)

#### Integrating MDSR into PHEM was a mixed blessing

An innovative feature of Ethiopia's MDSR system is that 1 year after its introduction, the FMOH decided to merge notification and reporting of maternal deaths into the national PHEM, a key function of the Ethiopian Public Health Institute (EPHI). Maternal death became the 21st mandatory reportable condition. Inclusion of maternal mortality within PHEM was seen as further proof of its prioritisation at high political level, making Ethiopia's MMR an official ‘emergency’.

Now MDSR is under the immediately and weekly reportable diseases, it is included as the 21st…The main thing is, MDSR is of public health importance when the government included it under surveillance. (Regional Health Bureau PHEM officer)

This policy, however, required integration across two vertical health programmes at every level. Responsibility for data collection, management and analysis shifted from the Ministry's MCH directorate to PHEM surveillance officers, although MCH experts were still expected to contribute to individual death reviews and interpretation of aggregated data; both teams were tasked with ensuring evidence-based actions resulted from the review process.

According to respondents, if MCH and PHEM directorates had a close working relationship, MDSR could flourish. In these cases, integration was seen to pool strengths from both teams and increase the likelihood of follow-up action.

The integration helps [us] to support each other because maternal and youth officers and PHEM officers are now giving [it] attention…There will be a common understanding and support to each other. (Zonal PHEM Officer, Amhara)

When a maternal death happens, both of us—surveillance expert and MCH expert—are involved in a meeting to give a professional explanation. The cause of the death, what action [should be] taken, where the problem happened? (District MCH officer, Tigray)

If, however, there were tensions over ownership or confusion as to which directorate should manage specific components (reporting suspected deaths, conducting verbal autopsies, arranging review committee meetings and taking responsibility for implementing identified responses), the process will tend to collapse.

There was a communication gap when we received the letter from Ministry of Health [about integration]. There was also the issue raised at district level that they didn't want to accept the activities due to [lack of] training. (Regional Health Bureau MCH team leader)

MDSR has its own procedure in writing a review…but in trying to do that [integrate with PHEM] there were challenges of not knowing what to do…There were difficulties in following procedures. (District Health Office MDSR focal person, Amhara)

Although respondents were interviewed over a year after formal MDSR–PHEM integration, there was still lack of clarity around how the two departments should work together. Interviewees highlighted that integration caused significant disruption to MDSR as training was rolled-out across the country to PHEM officers and a new Implementation Manual was developed, printed and distributed to them. Furthermore, there were no clear guidelines for how to build collaborative environments across the directorates, which were not always housed in the same buildings. There did not appear to be any precedent for sharing reporting across vertical programmes and thus whether respective MDSR focal persons were able to forge a productive working relationship depended on their personalities and motivation.

#### Resistance to MDSR and loss of momentum posed early threats

When asked about factors that impeded smooth introduction of MDSR, respondents mentioned widespread fear that an increase in reported maternal deaths could lead to legal or disciplinary actions. Health providers and administrators expressed concern that given the national focus on reducing the country's high maternal mortality, each maternal death could be interpreted as professional malpractice or negligence, or failure to comply with policy.

Concerns about potential negative repercussions were raised in all four study sites and among all levels and types of health professionals. These fears have persisted, despite emphasis throughout MDSR documents and training materials on avoiding individual blame:

Management [of facilities] looks at it from a negative point of view, that they will be held responsible for the maternal deaths. But instead of being held responsible, it should be looked at from a saving life perspective…(District Health Office MDSR focal person, Amhara)

There was the fear of being held responsible if a mother dies when under the care of a provider. (Health Centre Head, SNNPR)

We are teaching mothers to give birth at health centres, so if the mother delivers at home, [community members] think that they are accountable. So there is fear of blame. (Acting Health Centre Director, Tigray)

Such fears were not unfounded. Respondents narrated cases of clinicians and family members being detained by police investigating maternal deaths, although no one seems to have been arrested. Fear of repercussions was seen to dissuade accurate reporting of maternal deaths, and threatened the whole MDSR system.

Professionals are asking for legal cover…I suspect under-reporting will be due to this problem. (Zonal MCH team member, Oromia)

Fear of blame and legal measures following a maternal death partly resulted from the strong political will to tackle MMR. Several respondents highlighted that use of the slogan ‘No woman should die while giving life’ inadvertently frightened frontline providers and lower level public health officials. Although the slogan was formally abandoned during 2014, it remained printed on older documents and was well remembered by study participants for having both enhanced the impetus for using MDSR as a tool to reduce maternal mortality and simultaneously fostering reluctance to admit to maternal deaths at district and community levels.

The nascent MDSR system also suffered a drop in momentum due to frequent staff departures. Although the original wave of training in 2013 and follow-up roll-out to PHEM surveillance officers led to initial enthusiasm for initiating MDSR, trained individuals started to be reassigned or were absent for long periods of time due to new initiatives. High turnover and poor handover procedures meant many health facilities were left without staff who felt ownership over MDSR implementation and sometimes there was simply no one left who knew what the process required or where the requisite forms were kept.

…Attrition when somebody reallocates…for example the health centre head was one of the trained right…so when the head is changed they [other staff] become lenient and who would collect [data] and review [them]? He was the one we trained as a chairman. So they need to share skills. We are advocating for one person to pass the responsibilities to the next person when leaving; it is impossible to keep on training every new person. (Regional Health Bureau Staff)

The sense that MDSR was new and urgent also faded over time, and competing priorities increased the likelihood that staff would be reassigned. One unforeseen side effect of integrating maternal mortality into PHEM that worried respondents was that maternal deaths were not the same kind of emergency as an outbreak of measles or sudden cluster of polio cases. As PHEM teams took responsibility for MDSR, they risked being diverted to emerging health crises; in the first 2 years of MDSR in Ethiopia, Ebola preparedness, measles vaccination campaigns and drought relief work occupied PHEM teams and disrupted MDSR.

When it was said MDSR should be integrated, we assigned one ‘focal person’. But the focal person moved to…the Ebola response…because of this, no one is assigned for MDSR. (Regional Health Bureau staff)

There were few suggestions for how to address this. Respondents felt the inclusion of maternal mortality as a public health emergency was a valuable innovation within MDSR, and they trusted the broader national surveillance system to increase the chances that maternal deaths would be identified and reported upwards. Yet there was widespread acknowledgement that since maternal deaths were not contagious or ‘epidemic’ in the same way as cholera, for example, scarce resources would regularly be diverted elsewhere.

### Outcomes: What are the perceived effects of MDSR?

Respondents felt that MDSR had been in place long enough to enable reflection on its contribution to provision of MCH services. Positive changes resulting from MDSR were considered to be increased confidence in the data available on causes of maternal deaths, improved communication within the health system, and more appropriately targeted actions taken to reduce maternal mortality.

#### Streamlined information and communication

At aggregate level, district, zonal or regional committees were seen to have increased their capacity to assess local patterns in maternal mortality. MDSR provided a clear structure for how to discuss maternal deaths, including standardised guidance for identifying obstetric causes, social determinants and actions that might prevent the same chain of events occurring in future. Linking responses directly to every death gave staff confidence that they were acting appropriately, based on empirical evidence.

MDSR is a reason to study about maternal death. We identify, discuss, act and promise to prevent similar deaths, so all this is a change from MDSR. (District MCH team member, Tigray)

Similarly, in health facilities, while maternal death cases had previously been discussed at staff meetings, they were not always analysed systematically.

Before MDSR, maternal death was seen as any death, but now it is seen as a critical issue for discussion and it will get a solution soon. Now the awareness is good among physicians and midwives. There is good attention in reporting the maternal death and in dealing with the issue. Now the data are real data because we are taking the data from home through verbal autopsy. (Hospital matron, Oromia)

Two main benefits were perceived to result from MDSR data collection and reporting requirements. First, appreciation for having reliable data on which to act, has led to improvement in documenting and managing case notes in facilities. Filling out case notes accurately and the inclusion of more detailed information on the circumstances of each death are now seen to have a useful purpose rather than being an additional burden on staff workload.

Before, when a death occurred, they [hospital staff] do what needs to be done, but there was no record keeping. It has improved our medical recording. Before there were death summaries just for releasing the body but now you will find detailed summaries. (Hospital Director, Amhara)

Second, the availability of more detailed information strengthened communication across the health system, as well as between individual health providers, and between health authorities and communities. Hospital staff began to realise that they needed information from referring heath centres and health posts or from family members to understand why women arrived in an extremely critical state or already dead; health centres began relying more on existing ‘liaison officers’ to accompany referrals to hospitals so crucial information would be relayed to providers. Following review committee meetings, information was then fed down the chain to build closer relationships across institutions.

After establishment of MDSR committee we have a monthly meeting, discussion and mentor the health centres through the MDSR committee. MDSR…needs strong relations between Health centres and hospitals. (Hospital service provider, Oromia)

In some cases, district or zonal staff attended ‘catchment area meetings’ in order to ensure a comprehensive approach was taken for analysing available information, from the levels of awareness of pregnancy warning signs among community members, through referral pathways, transport infrastructure, and timely care provided at facilities.

We can know if the cause of a maternal death is related to shortage of health care providers, transportation, or provision of supply. It has its own way of doing analysis. That analysis will create ways that will eventually help in making decisions…(District health staff, Amhara)

#### More refined responses

From the inception of MDSR in Ethiopia, the ‘R’ was emphasised as the main purpose of the system. Study participants highlighted that the main goal of MDSR is to prevent future deaths, and described actions taken to address maternal risk factors raised through the review process.

Identification of measures put into place following maternal death reviews drew on the existing arsenal of FMOH MCH activities, including ‘community consultations’ and other health promotion approaches, ensuring ambulances were maintained and available, revising staff rosters to ensure midwives were on staff in facilities at all times, and updating protocols and updating provider skills. Respondents believed, however, that the MDSR process helped to tailor action plans to reflect what was learnt through analysing reported deaths. This was particularly clear for actions taken within facilities, where responses followed individual deaths and very specific changes to the way services were structured, staffed and managed could be made, and targeted community-based activities introduced into the catchment area.

After the establishment of MDSR, when maternal death occurs, we observe, assess each and every case, [determining] why the death occurs, when and where the problems occur, and who takes the responsibilities. After identifying and assessing the problems, we make decisions, take actions and take lessons to prevent future maternal death. (Hospital medical director, Oromia)

If there is death in the community we will go to the (MDSR) committee and conduct a study, then we will arrange meeting and we will discuss and we will respond. For example…[one] problem was [the deceased woman's] husband did not allow her to go to the health facility early, so we went and discussed. Then we called all fathers to participate in a health conference. They discussed the issue and it created awareness about the problems. (Acting health centre director, Tigray)

Public health officials often had fewer options at their disposal, depending on budget allocation to their level of the health system. While districts were responsible for ambulance deployment and maintenance, the role of the zonal health bureau is primarily coordination and ensuring standardised improvements across districts and facilities.

What is the main reason for that death? Is it due to supply, technical problem or other limitation? So after identification of the reason we set priority action. For example if the problem is due to supply, we try to improve the logistics and if the problem is skill gap, we propose training and try to close the gap. (Zonal staff member, Oromia)

To date, respondents felt that actions were more clearly linked to new data from MDSR at lower, grassroots levels, while higher up the system the focus remained on strengthening implementation of MDSR with little evidence of using aggregated data for planning and budgeting purposes.

There are needs to make evidence based decision specially at higher level: federal, regional, and zonal levels. One of the things that needs improvement and we didn't succeed was data has not reached from the lower to higher levels…[but] I think in the future this problem will be solved. (Regional PHEM team member, SNNPR)

…sitting at regional level we have not reached the conclusion that we were able to prevent further deaths…of course, response at facility level is a response of the region as well. What I believe is missing is that we need to conduct studies/research based on the reports and make a wider analysis…at regional level there is nothing we have clearly identified as effect or outcome of MDSR, only at facility level. (Regional Health Bureau MCH head)

There was also some frustration that the proportion of estimated maternal deaths identified through MDSR remained extremely low, thus making it challenging to use the database to guide policy.

There are still limitations. For example, there were only 30 maternal deaths reported with verbal autopsy this year. There is staff turnover and lack of awareness, and we have to work more than this. (Zonal MDSR focal person, Oromia)

There was optimism, however, that assuming the system's momentum could be sustained, the system would continue to develop and strengthen, ultimately providing a resource for evidence-based decision-making.

Since the data we get from the community are accurate, it is good for future reporting for the country instead of doing estimated work [DHS]. What we are getting directly from the community indicates where we are and where we are going. (Zonal PHEM focal person, Amhara)

## Discussion

This case study was conducted across four zones in Ethiopia and has identified several lessons that can be applied from the introduction of MDSR. First, MDSR needs to become ‘systematised’ within public health at all levels so it becomes embedded into routine practices and supervision mechanisms, receiving clear support from above and aligning well with existing strategies. Next, roles and responsibilities within MDSR need to be clearly defined so that all stakeholders understand their roles, responsibilities and potential benefits to their particular professional niche. While integration of maternal deaths into surveillance structures is a feasible model that signifies the prioritisation of maternal mortality and takes advantage of existing reporting mechanisms, it can also cause problems if maternal health expertise is housed outside a vertically organised surveillance system. Third, to ensure the system's sustainability, regular supervision and feedback is required so that use of data to improve conditions on the ground can motivate providers, health system staff at all levels and community members, and prevent fears about its negative repercussions.

### Systematising MDSR

The MDSR cycle of identifying, reporting, analysing and acting on maternal deaths needs to become routine practice rather than viewed as a stand-alone system. Ensuring synergies between the use of MDSR to identify appropriate MCH improvements and other strategic policy documents makes it easier to adopt MDSR processes within existing programmes and initiatives. Strong political will and support for MDSR in its early phase also sends a positive message on the importance of its implementation, but this may fade with time and emergence of competing issues.

In Ethiopia, respondents valued the links between MDSR and achieving MDG targets, and its inclusion within the primary guiding health policy framework, but they also highlighted implementation gaps on the ground related to staff turnover, inadequate skills sharing between trained staff and their colleagues, and delays in distributing guidelines and data collection forms. This was also found in a review of MDSR in Malawi, where the lack of forms and uncertainty on how to fill them out emerged as a significant challenge.^18^

With time and sustained support, data collection, reporting and analysis procedures should become more familiar, lessening the perception that MDSR can pose an additional burden. In other countries, once MDSR becomes embedded as routine practice, coverage improves and allows for use of aggregated data for decision-making even at regional and national levels, although this can take 5–10 years.^11^ For this to happen, however, requires proactive recognition of local resistance to complying with MDSR and careful consideration of how to balance political support for reducing maternal mortality with avoiding the creation of a culture of fear of blame and repercussions for ‘allowing’ maternal deaths to occur.

### Clarity of functions

While inclusion of maternal death as 1 of 21 mandatory reportable conditions was perceived as one of the greatest strengths of Ethiopia's MDSR, it also provoked concern. This was partly a result of integration being introduced almost a year following the launch of the national MDSR system, causing significant delays while training and tool distribution were restarted.

PHEM and MCH staff at all levels had to negotiate collaboration against the backdrop of the vertical organisation of Ethiopia's health sector. While some teams were able to operationalise MDSR across their areas of expertise effectively this depended on individual personalities and existing working relationships. Where MCH and PHEM staff had no prior experience of working together or were physically located in separate sites, there were longer term challenges to the system.

Framing maternal mortality as a ‘public health emergency’ requiring immediate reporting may be politically astute, but created a tendency for regular diversion of attention and resources during infectious outbreaks. Since data collection for this study was completed, however, this problem has been acknowledged and restructuring within EPHI is likely to lead to a separate directorate for the MDSR programme, with a dedicated team of 4–5 people.

### Maintaining motivation

The fact that MDSR was well aligned with national MCH goals instilled stakeholders with long-term commitment to the system and assured them of its sustainability. Whether or not MDSR was incorporated into supervision mechanisms affected the degree of support perceived by respondents and it was clearly important to them to witness consistent support throughout the public health hierarchy. Regular monitoring encouraged staff engaged in MDSR to maintain their efforts, avoid neglecting the cycle of data collection and analysis when other initiatives were introduced, and helped bridge implementation gaps, for example, due to staff change or shortages.

After 2 years, changes attributed to MDSR were improvements in data availability and quality, enhanced communication across health system levels, and selection of better refined MCH activities tailored in response to maternal deaths. These changes were most apparent within facilities, however, and at district level where primary and secondary care and community-based health promotion were more closely linked. Evidence of MDSR benefits were taking longer to materialise higher up the structure.

Zonal and regional respondents often did not see clear benefits of MDSR for their work, and were more likely to express disappointment that the system was slow to capture a significant portion of maternal deaths or provide data useful for higher level policy development. This suggests the importance of managing expectations and identifying ways to engage with zonal and regional staff. There will invariably be a time lag between the start of death notification and existence of a large enough database for meaningful analysis. Recent introduction of regional-level databases and data management workshops for combined MCH and PHEM teams at zonal and regional levels may help incentivise ongoing MDSR efforts.

### Study limitations

This was a small-scale case study, considering MDSR introduction in just four zones across an extremely large, geographically disparate and highly populated country. Respondents were sampled purposively to gain a mix of roles and efforts were made to include both high-performing and low-performing districts in each zone; nonetheless, the findings are indicative rather than representative of MDSR experiences and perceptions in the four largest regions of the country.

Interviews were conducted 2 years after MDSR began and not all the original staff members involved in its introduction remained in post. Not all respondents will have remembered details of their experiences in the early days of MDSR, leading to recall and social desirability bias. The latter was a particular risk as the assessment was conducted by E4A, a programme itself involved in the design of MDSR tools and training materials, although the interviewer himself had never worked in any of the sites.

To minimise inconvenience to participants, group interviews were offered to staff who shared an office or were working together at the time of data collection. Discussing the strengths and weaknesses of the programme in front of colleagues, especially if PHEM and MCH staff were interviewed together may have influenced both the dynamics of the conversation and the willingness of respondents to discuss certain challenges or interpersonal difficulties.

## Conclusion

Ethiopia is among the first African countries to introduce an MDSR system designed to collect data from community and facility settings. As the system is rolled out, lessons from early implementation in the four largest regions can help guide its expansion as well as provide insights to other countries introducing or strengthening similar systems.

In addition to building on MDSR's existing strengths such as its ‘fit’ within national health strategies, strong support throughout the health system and integration into the country's existing surveillance mechanisms, specific challenges will need to be addressed including better multisectoral cooperation and ensuring MDSR is systematised across training and supervision structures. Finally, as is true for many health policies or programmes, broader health system issues need to be addressed, including counteracting high staff turnover, strengthening available infrastructure and putting legal safeguards into place for health professionals.
