# Supplementary material for: ‘We identify, discuss, act and promise to prevent similar deaths’: a qualitative study of Ethiopia's Maternal Death Surveillance and Response system
Source: BMJ Glob Health. 2017 Mar 14;2(2):e000199. doi: 10.1136/bmjgh-2016-000199 (PMC5435261; doi:10.1136/bmjgh-2016-000199)
Supplement: supplementary file [file bmjgh-2016-000199supp.pdf]

Supplementary File: Semi Structured Interview Topic Guide: English Version

Areas of Inquiry	Specific Topics	Suggested Probes
**Introduction/ Background**	Please can you describe your current involvement in MCH in the zone and/or the region, with specific mention of how you are involved in the Maternal Death Surveillance and Response system?	• What are your roles and responsibilities?<br>• What are the main tasks that you conduct on a regular basis (every week or month)?
**Changes over time**	Have you observed any changes relating to maternal health over the past year or so?<br>What kinds of things are different and what kinds of things have stayed the same within your MCH work compared to about a year ago?	• Can you give examples of any changes related to awareness and policies on maternal health?<br>• Can you give any examples of changes in the quality of health services related to maternal health?<br>• Have these been positive or negative changes?
**Drivers of change**	What are the reasons behind any changes or development that you have observed in maternal health or provision of care?	• Why did the changes that you mentioned occur?<br>• For things that have stayed the same, why do you think there haven't been more changes?
**History of MDSR**	Please can you describe your experience of the introduction of MDSR and how it was implemented at the beginning. Try to	• What involvement did you have with MDSR training?

	remember exactly what happened at what time, and what your thoughts were about those events.	• How were the different components of MDSR established here, and what were initial reactions to it?
**Facilitators and barriers to MDSR introduction**	In your view, what kinds of factors here made MDSR easy or difficult to introduce into the existing health system?	• What were some of the early challenges when MDSR was first introduced in your area or facility?<br>• What conditions facilitated MDSR here?<br>• If you could go back in time, what would you do to make the introduction of MDSR better or easier?
**Integration of MDSR into PHEM**	As you know, maternal deaths are now one of the immediately notifiable events in PHEM reporting. How has this affected the MDSR system here?	• How has the MDSR process changed in your opinion since integration?<br>• What are the benefits or the risks of integrating PHEM and MDSR?
**Current MDSR functioning**	Please describe how you perceive the current MDSR system?<br><br>What do you think will happen to MDSR in the future and how can it be strengthened to ensure its sustainability?	• Which parts of MDSR are functioning and which still need to be implemented?<br>• Are there any gaps in how MDSR is being implemented, and if so, what do you recommend for addressing these?<br>• What is going well and what is going not so well?

**Effects of MDSR**	Do you think MDSR has had any noticeable impacts so far? Please name anything you feel is a result of MDSR, even if indirect.<br><br>After they have finished responding, probe for effects on any of the following:	Let the respondent think of examples.<br><br>• Has MSDR had any effects on *skills of health workers to recognise and manage obstetric emergencies*? If so, what have these been?<br>• Has MSDR had any effects on the availability of *essential drugs and equipment needed for maternity cases*? Please give examples<br>• Has MSDR has had any effects on *referrals or transportation*? If so, what has been the effect of the MDSR system?<br>• Have there been any changes in *communication between health workers or facilities* that can be linked to MDSR? Please describe. *What about between health services and community members?*
